# UBE2C functions as a potential oncogene by enhancing cell proliferation, migration, invasion, and drug resistance in hepatocellular carcinoma cells

**DOI:** 10.1042/BSR20182384

**Published:** 2019-04-17

**Authors:** Yu Xiong, Jing Lu, Qinliang Fang, Yuyan Lu, Chengrong Xie, Huita Wu, Zhenyu Yin

**Affiliations:** Department of Hepatobiliary Surgery, Zhongshan Hospital, Xiamen University, Fujian Provincial Key Laboratory of Chronic Liver Disease and Hepatocellular Carcinoma, Xiamen 361004, Fujian, P.R. China

**Keywords:** drug resistance, growth, metastasis, sorafenib

## Abstract

Hepatocellular carcinoma (HCC) is the third leading cause of cancer-related mortality worldwide. Recently, ubiquitin-conjugating enzyme E2C (UBE2C) has been reported to be overexpressed in human cancers and act as a potential oncogene. However, little is known about the functional roles of UBE2C in HCC progression. In the present study, analysis of UBE2C mRNA expression in The Cancer Genome Atlas (TCGA) dataset reveals that significantly higher UBE2C mRNA levels was found in HCC tissues and associated with higher HCC grade. Elevated UBE2C mRNA levels in HCC indicated worsened survival probabilities. Through performing loss-of-function assays, we demonstrated that knockdown of UBE2C expression obviously suppressed proliferation, migration, and invasion of HCC cells *in vitro*. Moreover, HCC cells with UBE2C knockdown showed higher sensitivity for the treatment of chemotherapeutic drug, including adriamycin (ADR) and 5-fluorouracil (5-FU). Silencing of UBE2C also increased the sensitivity of HCC cells to sorafenib, an approved treatment for patients with advanced-stage HCC. Our findings strongly suggest that UBE2C emerges as a marker for prognosis in HCC, and blocking UBE2C may be a novel strategy for HCC therapies.

## Introduction

Hepatocellular carcinoma (HCC) is the sixth most common cancer and the third leading cause of cancer-related mortality worldwide [1]. During the past two decades, the great advancement of HCC treatment has been achieved, including surgical resection, ablation, liver transplantation, and targetted therapy. However, the 5-year survival rate for HCC has remained unsatisfied at less than 12% due to tumor relapse and metastasis [2,3]. Therefore, to discover novel effective therapeutic treatments and to improve the prognoses of HCC patients, it will be essential to better understand the molecular mechanisms of HCC initiation and progression.

Ubiquitin-conjugating enzyme E2C (UBE2C) is a member of the E2 family and encoded by the *UbcH10* gene located on human chromosome 20q13.12, which catalyzes degradation of several target proteins. Interestingly, UBE2C is nearly undetectable in normal tissues, at both the mRNA or protein levels, while increasing evidence has shown that UBE2C expression is up-regulated in several human cancers, such as esophageal squamous-cell carcinoma, gastric cancer, and non-small cell lung cancer and rectal cancer [4–7]. UBE2C overexpression can enhance proliferation, invasion and cisplatin resistance, and inhibit apoptosis [7–9]. Moreover, UBE2C protects cancer cells from autophagic death [4,10]. However, its functional role in HCC carcinogenesis remains poorly defined.

In the present study using an *in silico* approach and public transcriptomic data, we revealed that deregulation of UBE2C expression were associated with worse outcome of HCC patients. Functional assays showed that knockdown of UBE2C expression dramatically inhibited proliferation, migration, and invasion. In addition, depletion of UBE2C decreased the sensitivity of HCC cells to the chemotherapeutic drugs and molecular-targetted agent sorafenib. Our results indicated that UBE2C may be a promising target for HCC treatment.

## Materials and methods

### Bioinformatics analysis

RNA-seq data of The Cancer Genome Atlas (TCGA) was analyzed. For the expression analysis, cases were classified into different group according to tumor grade. Cases were divided into two groups based on the average value of UBE2C mRNA expression, and then the prognoses of HCC patients with different levels of UBE2C mRNA expression were analyzed by the Kaplan–Meier survival curve.

### Cell culture

HEK-293T, SMMC-7721, and SK-Hep-1 cells were purchased from the Cell Bank of Chinese Academy of Sciences (China). Cells were cultured in DMEM (Hyclone) supplemented with 10% fetal bovine serum (FBS, Biological Industries), 100 U/ml penicillin, and 100g/ml streptomycin at 37°C with 5% CO_2_.

### Construct of stable cells with UBE2C knockdown

Two different shRNAs targetting UBE2C was inserted into lentiviral vector GV248 and constructed by Genechem Company (Shanghai, China). Scramble shRNA was taken as control (shNC). The target sequence of UBE2C shRNA was shown as follow: sh1: CCTGCAAGAAACCTACTCAAA, sh2: CCCTTACAATGCGCCCACAGT. Above lentiviral vectors were cotransfected with psPAX2 packaging plasmid and pMD2.G envelope plasmid into HEK-293T cells. After 48 h, the medium containing lentivirus were harvested and filtered. SK-Hep-1 and SMMC-7721 cells were infected with these lentiviral particles with 10 μg/ml polybrene. After 48 h, stable cells were selected by using 2 μg/ml puromycin for 1 week. The knockdown efficacy was detected by western blot.

### Cell viability detection

To detect the effect of UBE2C knockdown on cell proliferation, 2 × 10^3^ cells in 100 μl DMEM were seeded in 96-well plate. At different time point (1, 2, 3, and 4 days), cell proliferation was detected by Cell Counting Kit-8 (CCK-8, Dojindo, Beijing) assay. Total 10 μl of CCK-8 was added to each well. After 1.5 h, the absorbance was measured at a wavelength of 450 nm. To detect the effect of UBE2C knockdown on drug sensitivity, 2 × 10^3^ cells were seeded in 96-well plate and treated with adriamycin (ADR), 5-fluorouracil (5-FU), or sorafenib. All the drugs were purchased from Selleck company. After 48 h, cell viability was detected by CCK-8 as described above.

### Colony formation assay

The indicated cells (2 × 10^3^) were plated into a six-well plate. After being cultured for 2 weeks, the cell colony was fixed in methanol for 20 min at 25˚C and then visualized by crystal violet solution.

### Migration and invasion assay

For migration assay, 5 × 10^4^ cells suspended in serum-free DMEM were plated into the upper chamber (8-μm pore size, Costar), and DMEM supplemented with 10% FBS was placed into the lower chamber. For invasion assay, 1 × 10^5^ cells suspended in serum-free DMEM were plated into the upper chamber coated with Matrigel (8-μm pore size, BD Bioscience), and DMEM supplemented with 10% FBS was placed into the lower chamber. After 24 h, the cells that had invaded through the membrane to the lower surface were fixed with methanol and stained with Crystal violet. Cells on the lower side of the membrane were counted in five random fields under an Olympus light microscope.

### Western blot

Cells were lysed in RIPA buffer (Beyotime, Beijing, China). Proteins were then quantitated using a bicinchoninic acid (BCA) protein quantification kit (Thermo). A total of 30 μg protein sample were separated using 10% SDS-PAGE and transferred onto a PVDF membrane (Millipore). Then, the membrane was incubated with specific primary antibodies followed by their respective secondary antibodies. The bands were visualized by ECL Western Blotting Substrate (Millipore). Antibodies against UBE2C and β-actin were purchased from Proteintech (Wuhan, China).

### Statistics

Statistical analyses were done using GraphPad Prism 7. The Student *t* test or one-way analysis of variance followed by Dunnett’s multiple comparisons test were performed to compare the differences between two groups or more than two groups, respectively. Differences were considered statistically significant at the level of *P*<0.05.

## Results

### Overexpression of UBE2C predicts poor prognosis of HCC patients

**Figure 1:**
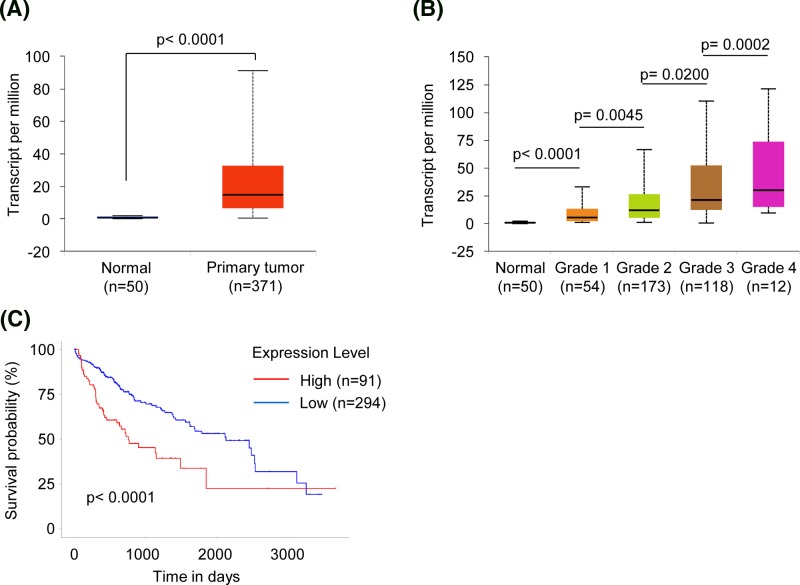
Overexpression of UBE2C mRNA is associated with malignancy in HCC (**A**) UBE2C mRNA expression in normal and HCC tissues from TCGA datasets. Data are mean ± S.D. (**B**) UBE2C mRNA expression in different grade HCC from TCGA datasets. Data are mean ± S.D. (**C**) Kaplan–Meier survival analysis of the whole HCC patients according to UBE2C mRNA expression.

In order to investigate the role of UBE2C in HCC tumorigenesis, we examined the HCC database of TCGA to evaluate the differential expression of UBE2C between normal and HCC tissues. As shown in Figure 1A, the mRNA expression of UBE2C was found to be significantly up-regulated in HCC compared with normal tissues. To identify potential associations between UBE2C mRNA expression and clinicopathological parameters, we compared its abundance amongst diverse subgroups based on tumor grade. We observed a significant increase of UBE2C expression in grade IV than grade I, II, and III (Figure 1B). Then, the Kaplan–Meier analysis was performed to assess the prognostic values of UBE2C mRNA expression in HCC. The results showed that HCC with higher expression levels of UBE2C was significantly correlated with the elevated rates of mortality (Figure 1C, *P*<0.0001). Taken together, these data strongly suggest that UBE2C is abnormally overexpressed in HCC patients and may serve as a new prognostic biomarker for HCC.

### Knockdown of UBE2C expression suppresses proliferation of HCC cells

**Figure 2:**
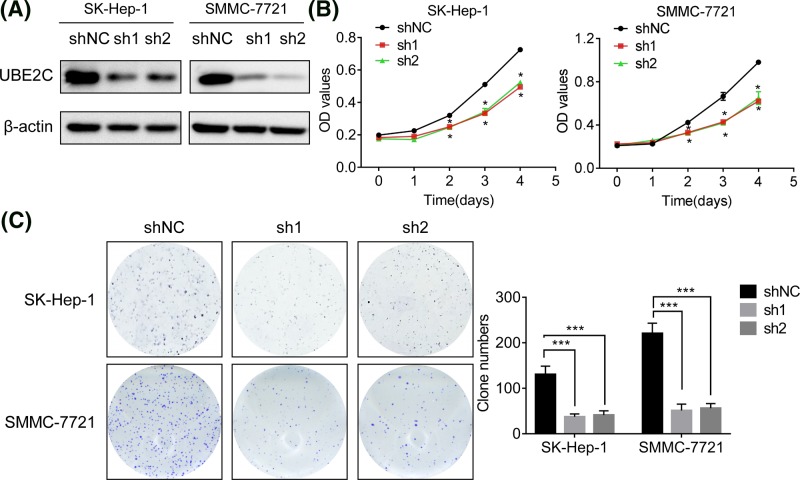
Knokcdown of UBE2C inhibits proliferation in HCC cells (**A**) SK-Hep-1 and SMMC-7721 cells were infected with lentiviral particles expressing UBE2C shRNA (sh1 and sh2) or the negative control (shNC). The effect of UBE2C shRNAs was confirmed by western blot analysis. (**B**) SK-Hep-1 and SMMC-7721 cells transfected with UBE2C shRNA or the negative control were cultured in a 96-well plate for 4 days. Cell proliferation was determined using the CCK-8 assay. (**C**) UBE2C depletion weakened the colony formation ability of both SK-Hep-1 and SMMC-7721 cells. Data are mean ± S.D. *P<0.05, ***P<0.0001.

To investigate the functional role of UBE2C on HCC progression, lentiviral particles expressing UBE2C shRNA (shUBE2C) were used to knockdown endogenous UBE2C in SK-Hep-1 and SMMC-7721 cells. Western blot assays showed that the protein expression levels of UBE2C were significantly reduced in both SK-Hep-1 and SMMC-7721 cells (Figure 2A). The effect of UBE2C knockown on HCC cell proliferation was determined by CCK8 assays, and the results revealed that UBE2C knockdown significantly supressed cell proliferation (Figure 2B). Similarly, colony formation capacity of SK-Hep-1 and SMMC-7721 cells was suppressed after the knockdown of UBE2C (Figure 2C). These data demonstrated that UBE2C can promote proliferation of HCC cells.

### Knockdown of UBE2C expression inhibits migration and invasion of HCC cells

**Figure 3:**
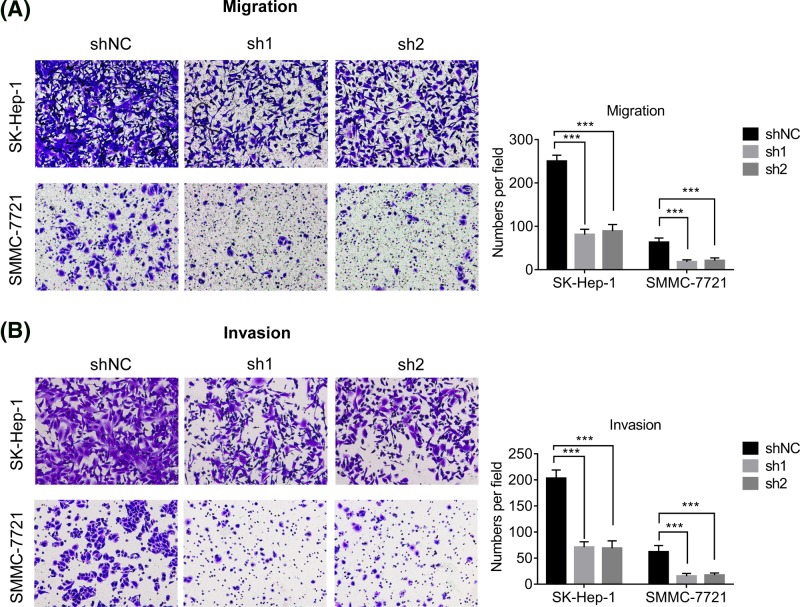
Depletion of UBE2C suppresses cell migration and invasion (**A, B**) The migration and invasive ability after knockdown of UBE2C in SK-Hep-1 and SMMC-7721 cells was assessed using transwell assays. Data are mean ± S.D. ***P<0.0001.

Cellular migration and invasion is critical for HCC metastasis. We then evaluated the effect of UBE2C on the migration and invasion of HCC cells. Transwell experiments demonstrated that knockdown of endogenous UBE2C expression apparently inhibited cell migration and invasion in both SK-Hep-1 and SMMC-7721 cells (Figure 3A,B).

### UBE2C decreases the chemotherapeutic sensitivity of HCC cells

**Figure 4:**
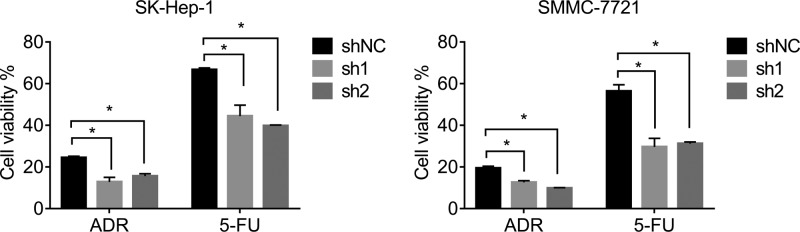
UBE2C decreases the chemotherapeutic sensitivity of HCC cells Control and UBE2C knockdown SK-Hep-1 and SMMC-7721 cells were treated with 5 μM ADR or 10 ug/ml 5-FU for 48 h. The cell viability was then detected by using CCK-8 assay. Data are mean ± S.D. *P<0.05.

A recent study demonstrated that UBE2C was involved in chemotherapeutic sensitivity in non-small cell lung cancer cells [9]. However, whether UBE2C regulates the chemotherapeutic sensitivity of HCC cells remains unknown. SK-Hep-1 and SMMC-7721 cells with down-regulation of UBE2C were treated with two different chemotherapeutic drug, ADR and 5-FU, for 48 h and then the cell viability was detected by CCK-8 assay. The results showed that UBE2C knockdown enhanced the sensitivity of both SK-Hep-1 and SMMC-7721 to ADR and 5-FU (Figure 4).

### UBE2C reduces the sensitivity of HCC cells to the molecular targeted agent sorafenib

**Figure 5:**
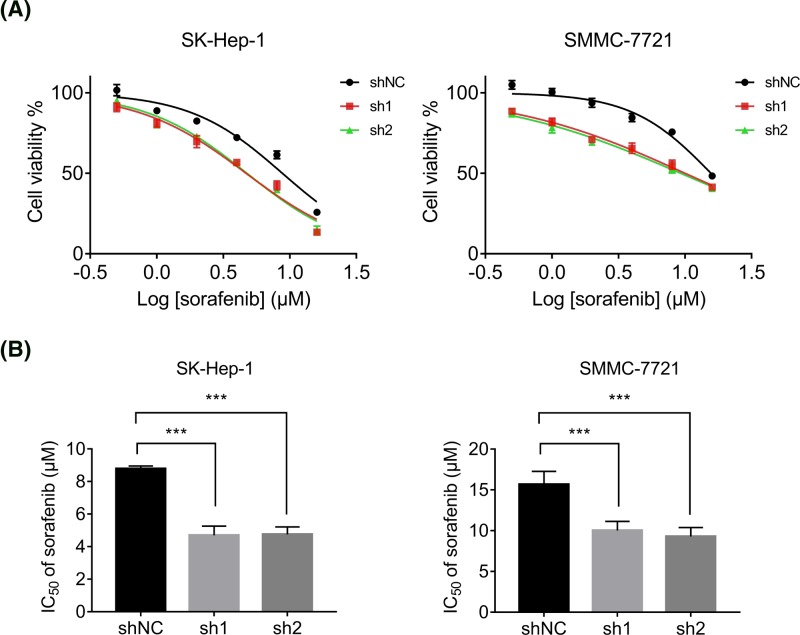
UBE2C reduces the sensitivity of HCC cells to the treatment of sorafenib (**A**) Control and UBE2C knockdown SK-Hep-1 and SMMC-7721 cells were treated with various concentrations of sorafenib for 48 h. The cell viability was then detected by using CCK-8 assay. (**B**) CCK-8 assay on control and UBE2C knockdown SK-Hep-1 and SMMC-7721 cells showed significant difference in IC_50_ of sorafenib. Data are mean ± S.D. ***P<0.0001.

Sorafenib is the first multikinase inhibitor that is the recommended treatment in advanced HCC [11]. To determine the influence of UBE2C in the sorafenib sensitivity of HCC, we compared the efficacy of sorafenib administration on SK-Hep-1 and SMMC-7721 cells with and without UBE2C knockdown. The cell viability was detected by CCK8 assay 48  h after the treatment of various concentration of sorafenib, and we found that depletion of UBE2C could decrease the cell viability of both SK-Hep-1 and SMMC-7721 cells (Figure 5A). The IC_50_ value of sorafenib on SK-Hep-1 and SMMC-7721 was significantly decreased after silencing the UBE2C expression (Figure 5B). Thus, we concluded that UBE2C was associated with sorafenib resistance.

## Discussion

Overexpression of UBE2C was found in various human cancers. In the present study, we further depicted the UBE2C mRNA expression pattern in normal and HCC tissues from TCGA database and found a significant up-regulation of UBE2C mRNA levels in HCC tissues. Moreover, higher UBE2C expression was also associated with advanced tumor grade of HCC and predicted worse clinical outcomes for HCC patients. Consistent with our findings, a previous study also demonstrated that up-regulation of UBE2C mRNA expression was frequently observed in HCC tissues and associated with tumor invasion, de-differentiation, and poor prognosis [12]. Some research has focussed the molecular mechanism of UBE2C deregulation in cancer. For example, miR-495 inhibits UBE2C expression through direct interaction with 3′-UTR of UBE2C mRNA [9]. Other miRNAs, including miR-17, miR-20, and miR-196a, are involved in UBE2C deregulation [13,14]. Moreover, UBE2C expression is also regulated by transcription factors. Transcription factor FoxM1 positively regulates UBE2C expression to protect glioma cells from autophagic death. FOXM1 binds to UBE2C promoter region and transcriptionally activates it, leading to UBE2C up-regulation [15,16]. UBE2C is transcriptionally activated by the mutant p53, while repressed by wild-type p53 [17]. Additionally, UBE2C is epitranscriptionally stabilized with maintenance of lower m^6^A level within its mature RNAs due to the up-regulation of m^6^A demethylase ALKBH5 [4]. However, to date, the underlying mechanism of UBE2C deregulation in HCC remains unknown, which needs further investigation.

The oncogenic effect of UBE2C in several human cancers has been revealed. In specific, depletion of endogenous UBE2C markedly reduces the level of phosphorylation AURKA via Wnt/β–catenin and PI3K/Akt signaling pathways and then suppressed the growth and metastasis of gastric cancer [8]. UBE2C selectively represses autophagic death and disruption of UBE2C-mediated autophagy repression attenuates cell proliferation, clonogenicity, and invasive growth in non-small cell lung cancer (NSCLC) cells [9]. Zhang et al. also demonstrated that UBE2C regulated miR-381 expression to promote cell proliferation and invasion in rectal carcinoma cells [7]. Besides, the underlying mechanism of UBE2C in tumorigenesis and progression remains largely unknown. Here, our data showed that knockdown of UBE2C significantly attenuated cell proliferation, migration, and invasion in HCC cells *in vitro*, further supporting that UBE2C functions as a bona fide oncogene in human cancers. However, the exact mechanism of UBE2C in HCC progression has not been elucidated. A previous study using transcriptomic functional annotation and protein–protein interacting network analyses demonstrated that the histone-lysine N-methyltransferase (EZH2) and UBE2C were identified as principal interacting proteins of druggable networks [18], implying that the EZH2–UBE2C interaction may be critical for cancer progression.

Increase evidence proved that UBE2C represents a potential candidate biomarker, whose expression levels could be employed to predict response or resistance to chemotherapy or targeted agents. In colorectal cancer, knockdown of UBE2C sensitized the cells to pharmacological treatments with irinotecan, SN-38 and cetuximab partly through down-regulation of AKT [19]. In breast cancer, UBE2C knockdown increased the sensitivity of cancer cells to epirubicin and docetaxel and promoted the apoptosis induced by these two drugs through impaired the increased BCL-2 and MDR-1 expression levels [20]. Similarly, our findings also demonstrated that UBE2C regulated chemotherapeutic sensitivity of HCC cells. Sorafenib is the only approved drug for advanced-stage HCC. Sorafenib treatment suppresses HCC cell proliferation and angiogenesis through the suppression of Raf kinase and the inhibition of vascular endothelial growth factor receptor (VEGFR) [21]. However, only 30–40% patients were sensitive to sorafenib, and some patients developed drug resistance after several months of sorafenib treatment [22]. Interestingly, our present study is the first to show the effect of UBE2C on the sensitivity of HCC cells against sorafenib treatment. This point, which is shown in a scheme but needs to be further explored, provides a theoretical evidence for our future research.

In summary, we show that depletion of UBEC2 impairs HCC cell proliferation, migration invasion, and drug resistance *in vitro*. Our findings indicate that UBE2C may be a promising therapeutic target for HCC treatment.

## Abbreviations

5-FU: 5-fluorouracil
ADR: adriamycin
FBS: fetal bovine serum
HCC: hepatocellular carcinoma
EZH2: enhancer of zeste homolog 2
TCGA: The Cancer Genome Atlas
UBE2C: ubiquitin-conjugating enzyme E2C
